# The prognostic value of HGF-c-MET signaling pathway in Gastric Cancer: a study based on TCGA and GEO databases

**DOI:** 10.7150/ijms.44952

**Published:** 2020-07-19

**Authors:** Chao Wang, Wenqi Xi, Jun Ji, Qu Cai, Qianfu Zhao, Jinling Jiang, Chenfei Zhou, Min Shi, Huan Zhang, Zhenggang Zhu, Jun Zhang

**Affiliations:** 1 Department of Oncology, Ruijin Hospital, Shanghai Jiao Tong University School of Medicine, No. 197 Ruijin er Road, Shanghai, 200025, China.; 2 Shanghai Institute of Digestive Surgery, Ruijin Hospital, Shanghai Jiao Tong University School of Medicine, No. 197 Ruijin er Road, Shanghai, 200025, China.; 3 Department of Radiology, Ruijin Hospital, Shanghai Jiao Tong University School of Medicine, No. 197 Ruijin er Road, Shanghai, 200025, China.

**Keywords:** HGF, c-MET, gastric cancer, TCGA, prognosis, GEO

## Abstract

Gastric cancer is a heterogeneous tumor that underlying molecular mechanisms are largely unclear. This study aimed to elucidate the expression level of HGF-c-MET in gastric cancer patients and to investigate the prognostic and diagnostic value of HGF-c-MET. *In silico* analysis of the TCGA and GEO database found that HGF and c-MET mRNA expression are significantly higher in gastric cancer tissues than those in peritumor tissues. Both higher mRNA expression of HGF and c-MET were associated with a poorer prognosis. c-MET expression was modulated by methylation in the promoter regions. HGF was positively correlated with CD8+ T cell, CD4+ T cell, macrophage, neutrophil and dendritic cell. Furthermore, functional enrichment analysis and protein-protein interaction networks further shown that HGF-c-MET and related proteins mainly participated in growth factor receptor binding, protein tyrosine kinase activity and signaling receptor binding. Finally, outcome of GSEA analysis showed 13 shared KEGG pathways enriched in high expressed group of HGF and c-MET.

## Introduction

Among the most common cancers in the world, gastric cancer (GC) ranked the third in terms of cancer incidence and fifth in terms of mortality 1, which seriously threatened the health of citizens and increased tight pressure and heavy burden on families and society. While the mechanism of gastric cancer tumorigenesis was still ambiguous, it is urgent to discover the molecular and cellular mechanism of gastric cancer.

Our prior studies had found multiple genes and signaling pathways participated or play dominant role in tumorigenic process of gastric cancer, such as HGF, which secreted by cancer-associated fibroblasts, could promote vascularization in gastric cancer via activating PI3K/Akt and ERK1/2 signaling pathway 2, and accelerated proliferation, migration and invasion of MET-unamplified gastric cancer cells through activating c-MET/Stat3/twist1 signaling pathway 3. It was noteworthy that HGF-c-MET signaling pathway is activated in a range of cancer types including gastric cancer, colorectal cancer, head and neck cancer, hepatocellular carcinoma, lung cancer, urothelial carcinoma of bladder, glioblastoma, melanoma and ovarian cancer, among others 2, 4-11, and associated with an aggressive phenotype and poor prognosis in pancreatic cancer 12.

The gene, which encoded hepatocyte growth factor (HGF, also known as scatter factor), was located on chromosome 7, and have been composed of 17 introns and 18 exons 11, HGF was a pleiotropic cytokine initially synthesized and secreted as an inactive precursor (pro-HGF), and then converted into an active 90 kDa heterodimer composed of an α chain and a β chain. The α chain, which existed in mature HGF, possessed an N-terminal hairpin loop and four kringle domains (K1-K4), while the β chain contained a serine protease-like domain 13. HGF owned two c-MET binding sites, a high affinity site in the N-terminal and first kringle domains and a low-affinity site in the serine protease homology region 14. The c-MET was encoded by the proto-oncogene MET and synthesized as a 170 kDa single chain precursor protein (pro-c-MET), which followed by processing to the mature form by post-translational modifications. The mature c-MET had an extracellular α-subunit and a single-pass transmembrane β-subunit, which formed a disulfide-linked heterodimer. The β-subunit not only formed an extracellular ligand-binding domain, but also formed part of the intracellular region, which is responsible for kinase activity and regulation of effector signaling. All of them guaranteed c-MET to serve as a receptor tyrosine kinase (RTK) 15.

Our integrative analyses of HGF-c-MET signaling pathway in human gastric cancer patients based on public cancer databases (TCGA and GEO), aiming to better define the characteristics, extent and prognostic prediction of HGF-c-MET signaling pathway involved in gastric cancer.

## Materials and methods

### Data acquisition and characteristics

#### Data acquisition

The raw and processed data were acquired from The Cancer Genome Atlas (TCGA) in January 2016. The mRNA expression data and clinical information were downloaded and the following samples were excluded: (1) “0” gene expression values and (2) incomplete survival information and pathological characteristics. These processes were performed in R, using “RTCGA Toolbox” R packages, besides, the differential mRNA expression level of HGF and c-MET between gastric cancer tissues and normal tissues were obtained from the TCGA and GEO database (GSE27342) and were analyzed via the UALCAN web tool (http://ualcan.path.uab.edu/) 16.

### The Human Protein Atlas (HPA)

The representative immunohistochemistry images of HGF and c-MET in gastric cancer tissues of the same patient (ID: 2196) were downloaded from the Human Protein Atlas (HPA) (http://www.proteinatlas.org/) 17.

### The Kaplan-Meier plotter

The relationship between HGF, MET and the prognosis of gastric cancer patients were studied using the GEO database 18, which contained GSE14210, GSE15459, GSE22377, GSE29272, GSE51105 and GSE62254 and TCGA database 19. The hazard ratio with 95% confidence intervals and log rank *p*-value were also calculated.

### Identifying the related factors interacted with HGF and MET

The STRING analysis tool was applied to identify the interacting factors using HGF and c-MET as the query and homo sapiens were chosen as organism (https://string-db.org/), furthermore, the relationship between HGF/MET and related factors were evaluated using spearman correlation analysis, finally, the seven related factors included HGF/MET were uploaded into Database for Annotation, Visualization, and Integrated Discovery (DAVID) web tool (https://david.ncifcrf.gov/home.jsp) 20, Go function enrichment analysis and Kyoto Encyclopedia of Genes and Genomes (KEGG) pathway enrichment were performed. *P* value <0.05 was set as the cutoff criteria.

### Analysis of gastric cancer data in cBioportal for Cancer Genomics database

Both HGF and MET genes were analyzed by cBioportal data, which is an open-access downloaded bio-database. The primary search parameters included alterations (Mutation, amplification, mRNA high, deep deletion, multiple alterations) and predicted mutation location with the default setting across samples curated from stomach adenocarcinoma (TCGA PanCancer Atlas).

### Methylation status of HGF and MET

The mRNA expression and methylation status of promoter of HGF and MET were analyzed using the MethHC web tool (http://methhc.mbc.nctu.edu.tw/php/index.php) 21.

### The relationship between HGF/MET and immunocytes

The Tumor Immune Estimation Resource (TIMER) web tool (https://cistrome.shinyapps.io/timer/) was used to investigate the correlation between HGF/MET and major immunocytes 22.

### Gene set enrichment analysis (GSEA)

GSEA (http://software.broadinstitute.org/gsea/index.jsp) was utilized to explore biological function of HGF/MET genes 23. Annotated gene sets c2.cp.kegg.v5.2.symbols.gmt was selected as the reference gene sets. The expression level of HGF/MET were chosen as a phenotype label. The false defection rate q value <0.05 and normalized enrichment score (NSE) >1 were selected to sort the pathways enriched in each phenotype.

### Statistical analysis

We defined 1.09 and 4.22 as the cutoff value of HGF and MET for high and low expression according to the analysis of X-tile software based on their relationship with overall survival, respectively. Univariate Cox regression analysis was investigated to identify independent prognostic variables based on HGF level, MET level and other clinical characteristics, including age, gender, AJCC Stage, T classification and lymph node metastasis. *P* < 0.05 was considered as the cutoff p value to select the analyzed factors from the univariate analysis data to perform the multivariate model. A forward stepwise Cox regression model was chosen to select independent prognostic factors. The nomogram was constructed using the data of HGF, MET, Age, AJCC Stage and the package of rms in R version. The predictive accuracy of the nomogram was checked by concordance index (C-index) and assessed by calibration comparing nomogram-predicted with observed Kaplan-Meier estimates of survival probability, the C-index was positively related with the prognostic prediction. Differences between groups were compared by unpaired Student's *t*-test, Mann-Whitney *u*-test or one-way analysis of variance (ANOVA). Data were described as mean and standard error of the mean (SEM). All statistical analyses were executed using statistical programming language R for windows (cran.r-project.org). Two-tailed P-value less than 0.05 was set as statistically significance.

## Results

### HGF and c-MET expression were up-regulated in gastric cancer

**Figure 1:**
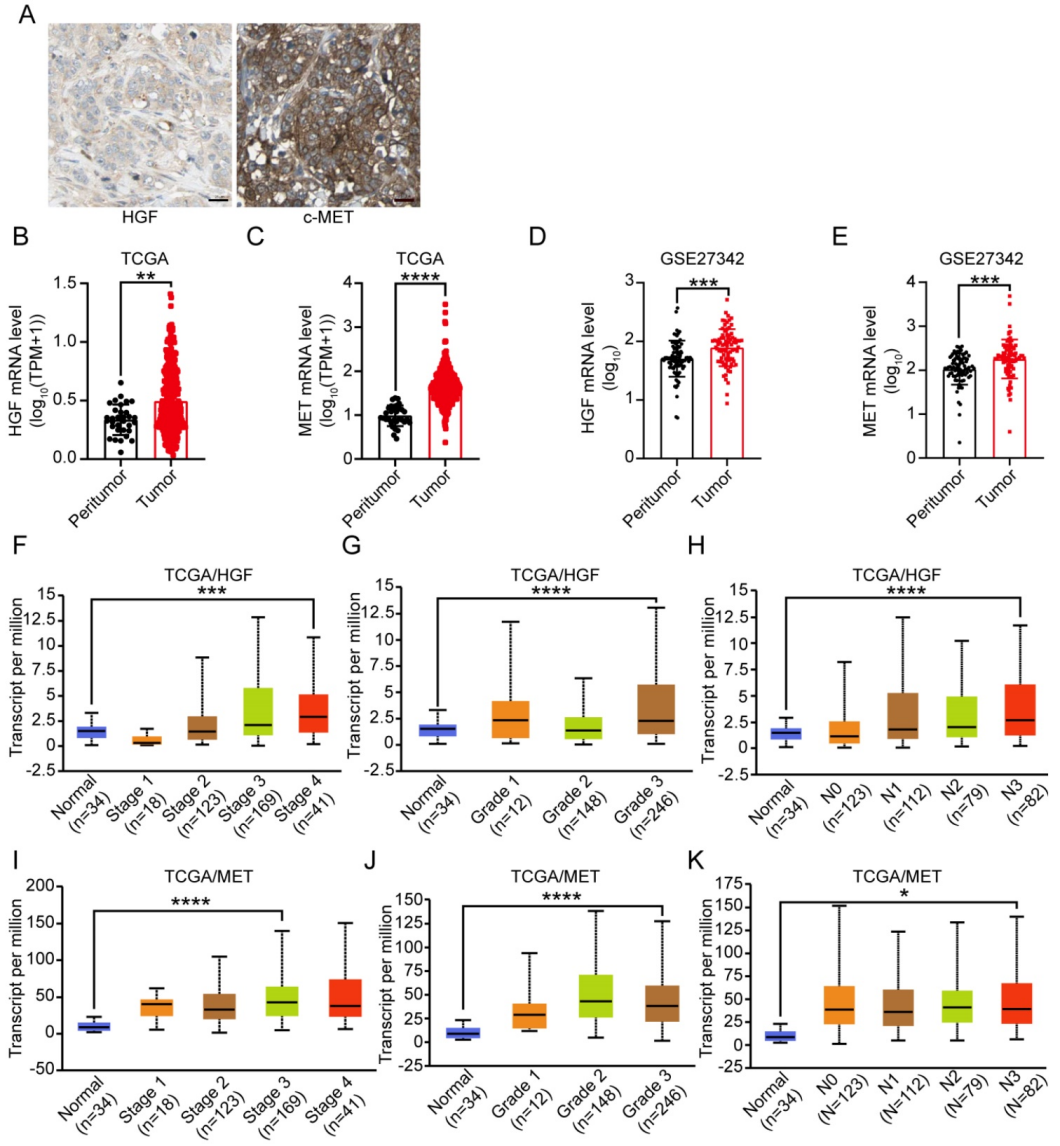
** Expression level of HGF and c-MET in gastric cancer. A.** Representative images from gastric cancer tissue serial sections stained with HGF and c-MET, which accessed from HPA database, the scale bar, 25 µm. **B-E.** HGF and c-MET mRNA expression were remarkably overexpressed in gastric cancer tissues compared with peritumor tissues in TCGA and GSE27342 database. **F.** The mRNA expression level of HGF in different AJCC stages based on TCGA database. **G.** The mRNA expression level of HGF in different tumor grades based on TCGA database. **H.** The mRNA expression level of HGF in different lymph node metastasis based on TCGA database. **I.** The mRNA expression level of c-MET in different AJCC stages based on TCGA database. **J.** The mRNA expression level of c-MET in different tumor grades based on TCGA database. **K.** The mRNA expression level of c-MET in different lymph node metastasis based on TCGA database. *, *P <*0.05; **, *P <*0.01; ***, *P <*0.001; ****, *P <*0.0001, respectively.

To analyze the protein expression of HGF and c-MET in gastric cancer, we searched The Human Protein Atlas (HPA) database and found that HGF and c-MET are highly expressed in the cytoplasm and plasma lemma of the gastric cancer cells belonged to the same patients (the images derived from the Human Protein Atlas (HPA) (http://www.protein atlas.org/)) (Figure 1A). Next step, the public cancer database (TCGA and GEO) were utilized to analyze the HGF and c-MET mRNA expression in peritumor and tumor tissues of gastric cancer patients, and the HGF and MET were both high-expressed in tumor tissues rather than those in peritumor ones (HGF: TCGA: *P <*0.01 and GSE27342: *P <*0.001; MET: TCGA: *P <*0.0001 and GSE27342: *P <*0.001; Figure 1B-E). Furthermore, gastric cancer samples with HGF and MET expression data across all patient characteristics were investigated from TCGA database, as shown in Figure 1F-K, the mRNA expression of HGF and MET were elevated obviously in highest AJCC stages, tumor grades and lymph node metastasis when compared with those in normal tissues (HGF: AJCC stage 4: *P <*0.001, tumor grade 3: *P <*0.0001, lymph node metastasis 3: *P <*0.0001; MET: AJCC stage 3: *P <*0.0001, tumor grade 3: *P <*0.0001, lymph node metastasis 3: *P <*0.05).

### Prognostic role of HGF and MET gene expression in gastric cancer patients

**Figure 2:**
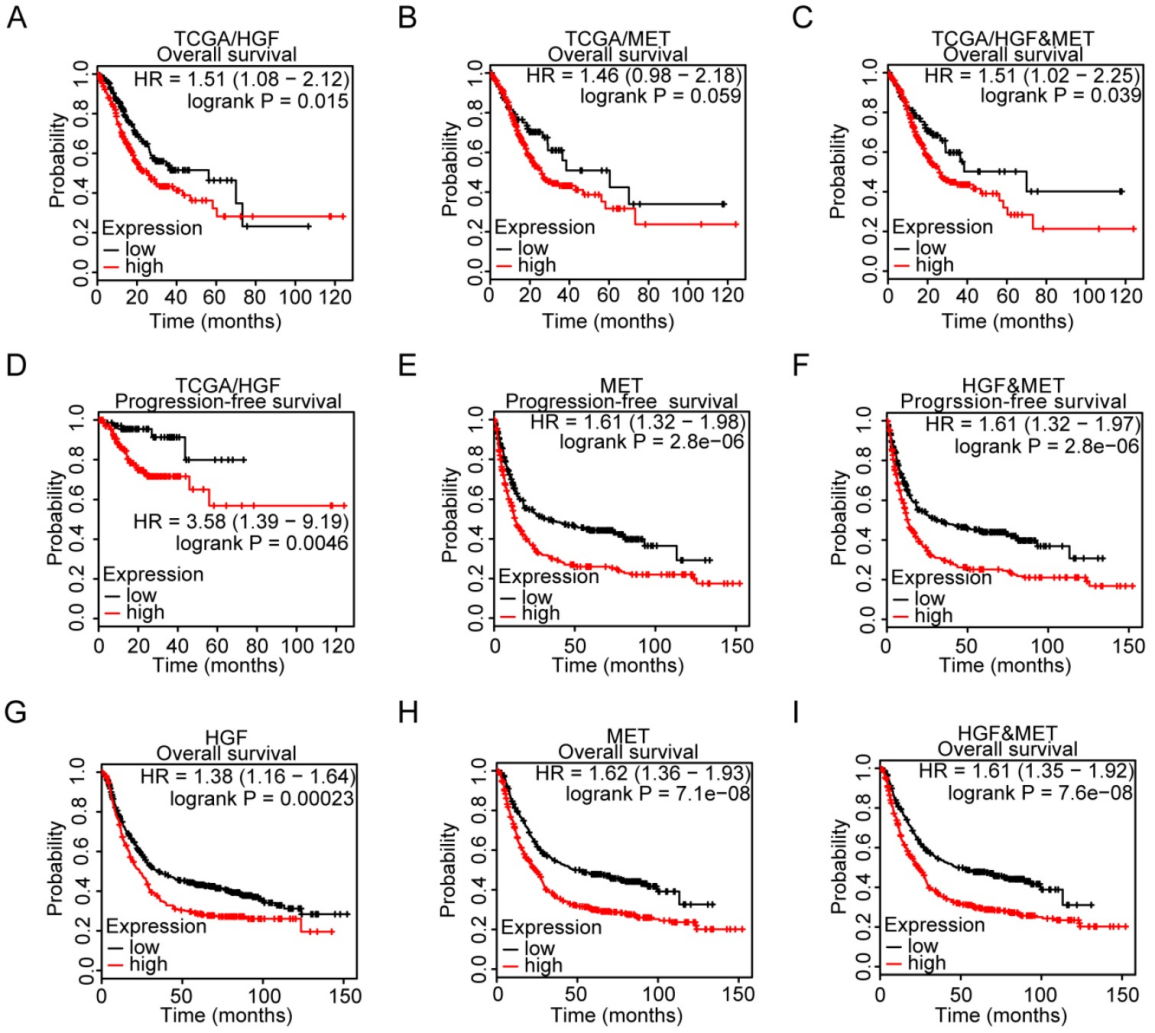
** High expression level of HGF and c-MET were indicators of unfavorable clinical outcome in gastric cancer. A.** Overall survival of patients in HGF-low expression group and HGF-high expression group based on TCGA database. **B.** Overall survival of patients in c-MET-low expression group and c-MET-high expression group based on TCGA database. **C.** Overall survival of patients in both of HGF and c-MET low expression group and both of HGF and c-MET high expression group based on TCGA database. **D.** Progression-free survival of patients in HGF-low expression group and HGF-high expression group based on TCGA database. **E.** Progression-free survival of patients in c-MET-low expression group and c-MET-high expression group based on GEO database. **F.** Progression-free survival of patients in both of HGF and c-MET low expression group and both of HGF and c-MET high expression group based on GEO database. **G.** Overall survival of patients in HGF-low expression group and HGF-high expression group based on GEO database. **H.** Overall survival of patients in c-MET-low expression group and c-MET-high expression group based on GEO database. **I.** Overall survival of patients in both of HGF and c-MET low expression group and both of HGF and c-MET high expression group based on GEO database. The survival time of patients was compared between groups using the Mantel-Cox test.

To explore the relationship between HGF and c-MET mRNA expression and prognosis of gastric cancer patients, the TCGA database was utilized and results demonstrated that HGF high expressed, and both HGF and MET high expressed gastric cancer patients have relatively shorter overall survival time, though the prognostic value of single marker “MET” in gastric cancer patients was marginal significant (TCGA/HGF: HR = 1.51 (1.08-2.12), *P* = 0.015, TCGA/MET: HR = 1.46 (0.98-2.18), *P* = 0.059, and TCGA/HGF&MET: HR = 1.51 (1.02-2.25), *P* = 0.039; Figure 2A-C). In terms of progression-free survival, HGF high expressed, MET high expressed, and both HGF and MET high expressed gastric cancer patients have less optimistic prognosis (TCGA/HGF: HR = 3.58 (1.39-9.19), *P* = 0.0046, MET: HR = 1.61 (1.32-1.98), *P* <0.0001, and HGF/MET: HR = 1.61 (1.32-1.97), *P* <0.0001, Figure 2D-F). To validate the prognostic value of HGF and MET, the GEO database, which contained GSE14210, GSE15459, GSE22377, GSE29272, GSE51105 and GSE62254 datasets, was determined to be validating cohort and results found that HGF high expressed, MET high expressed and both HGF and MET high expressed gastric cancer patients have significant shorter overall survival time than the low expressed groups, respectively (HGF: HR = 1.38 (1.16-1.64), *P* = 0.00023, MET: HR = 1.62 (1.36-1.93), *P* <0.0001, and HGF&MET: HR = 1.61 (1.35-1.92), *P* <0.0001; Figure 2G-I).

### Gene alteration analysis of HGF and c-MET by cBioportal

**Figure 3:**
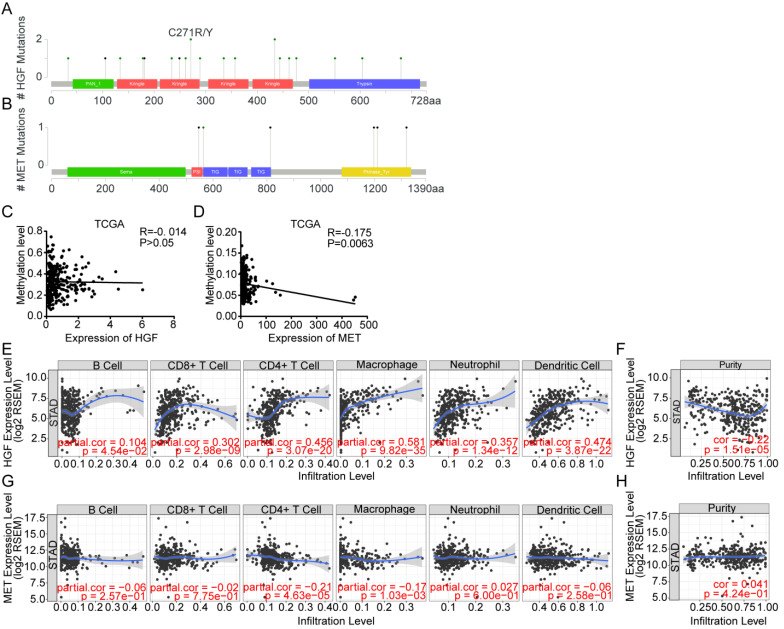
** Gene alteration analysis of HGF and c-MET by the cBioportal webtool. A.** screenshot of HGF mutation frequencies from the cBioPortal website. **B.** screenshot of c-MET mutation frequencies from the cBioPortal website. **C.** The correlation between expression of HGF and methylation level of HGF promoter in TCGA database. **D.** The correlation between expression of c-MET and methylation level of c-MET promoter in TCGA database. **E&F.** Correlation between HGF, infiltrated immune cells and tumor purity in gastric cancer. **G&H.** Correlation between c-MET, infiltrated immune cells and tumor purity in gastric cancer.

The cBioportal Web tool was utilized to explore HGF and c-MET gene alterations in gastric cancer. The mutation of HGF and mRNA upregulation of c-MET were the most common type of HGF and c-MET gene alterations in gastric cancer database (TCGA PanCan 2018), respectively. HGF mutation mainly existed in the Kringle domain of HGF, the 1 hot spot (C271R/Y) represent the commonly known mutations in the Kringle domain of HGF (Figure 3A&B). Besides, the methylation level and mRNA expression of HGF and c-MET in TCGA gastric cancer database were analyzed by the MethHC web tool, and results demonstrated that hypermethylation of promoter could decrease the c-MET mRNA expression with exception of HGF (c-MET: R=-0.175, *P =*0.0063; HGF: R=-0.014, *P >*0.05; Figure 3C&D).

### HGF was correlated with multiple immune cells

The microenvironment of tumor was composed of tumor cells, stromal cells, immune cells and numerous small molecular factors secreted by above cells, such as growth factors, chemokines, and so on. All components work together in the microenvironment and affect the tumorigenesis of gastric cancer, such as HGF which could not only be autocrine by tumor cells themselves but also participated in paracrine communication. Thus, the correlation between infiltrated immune cells and HGF was analyzed by TIMER, a web tool for researching infiltrated immune cells in the TCGA database, and results found that HGF have a negative correlation with the purity of gastric cancer and are positively correlated with CD8+ T cell, CD4+ T cell, macrophage, neutrophil and dendritic cell (tumor purity: cor = -0.22, P <0.0001, CD8+ T cell: partial.cor = 0.302, P <0.0001, CD4+ T cell: partial.cor = 0.456, P <0.0001, macrophage: partial.cor = 0.581, P <0.0001, neutrophil: partial.cor = 0.357, P <0.0001, dendritic cell: partial.cor = 0.474, P <0.0001; Figure 3E&F), While c-MET was negatively correlated with CD4+ T cell and macrophage cell (CD4+ T cell: partial.cor = -0.21, P <0.0001, macrophage cell: partial.cor = -0.17, P = 1.03e-03; Figure 3G). The positive correlation of tumor purity and c-Met expression demonstrated the tumoral expression of c-Met unlike a microenvironmental expression of HGF that has a negative association with tumor purity (cor = 0.041, P = 4.24e-01; Figure 3H).

### Protein-protein interactions among HGF-c-MET

**Figure 4:**
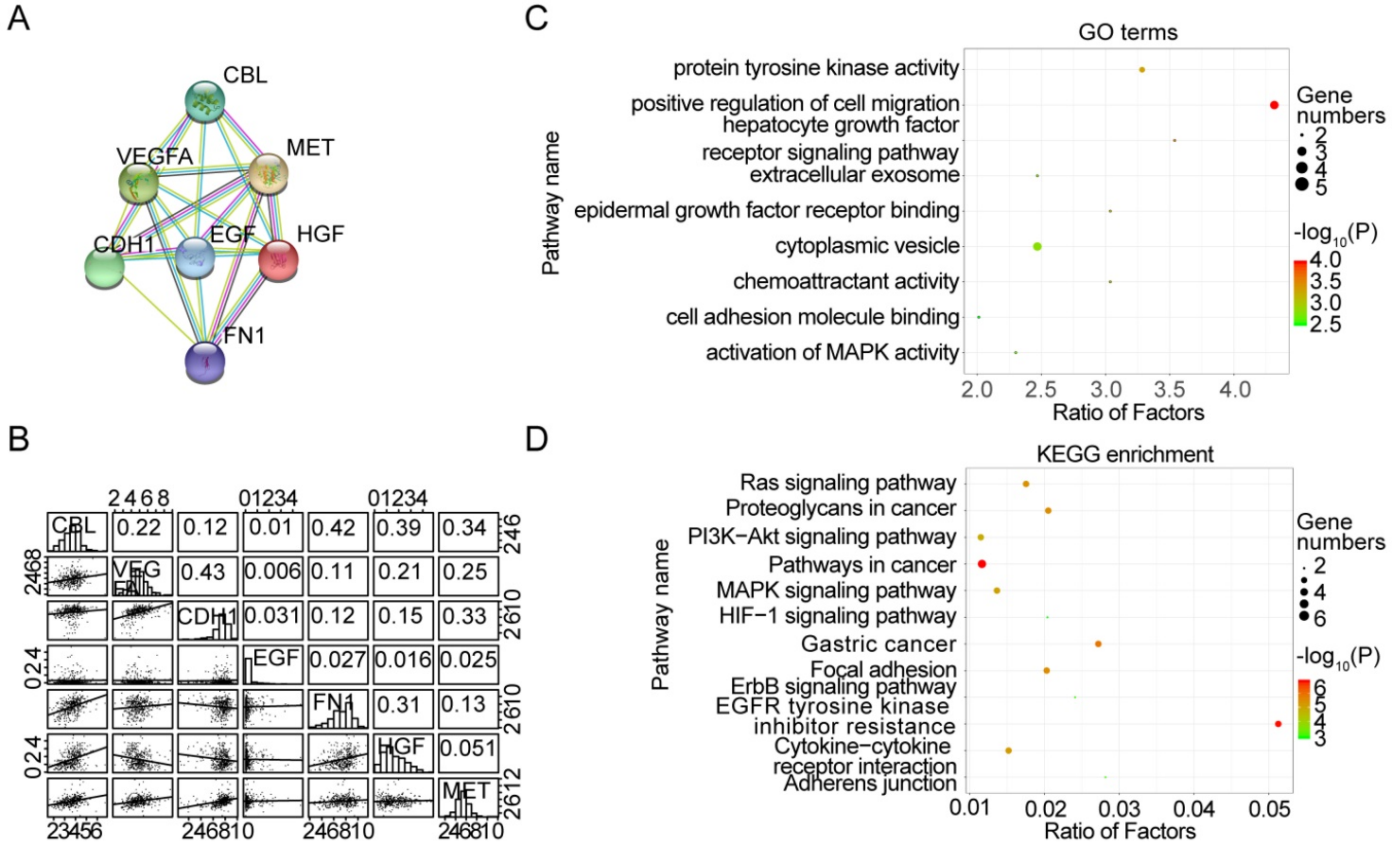
** Protein-protein interactions among HGF-c-MET. A.** the protein-protein interactions (PPI) network reflected the relationship between CBL, VEGFA, MET, HGF, EGF, CDH1 and FN1. **B.** Correlations among CBL, VEGFA, MET, HGF, EGF, CDH1 and FN1 levels in human gastric cancer tissues (TCGA, n=375). **C.** Top 9 Gene Ontology (GO) pathways with P value <0.05, -log_10_ (P value) >1.301. **D.** Top 12 KEGG pathways with P value <0.05, -log_10_ (P value) >1.301.

To better understand the interplay among the HGF and c-MET, we have analyzed protein-protein interaction (PPI) networks using STRING tool. The network was composed of 7 nodes, which are CBL, VEGFA, MET, EGF, CDH1, HGF and FN1 (Figure 4A), and the relationship between mRNA expression of 7 nodes was examined using TCGA database. HGF were positively correlated with CBL (R = 0.39, *P <*0.0001) and FN1 (R = 0.31, *P <*0.0001), and negatively correlated with VEGFA (R = 0.21, *P <*0.0001) and CHD1 (R = 0.15, *P <*0.01), besides, c-MET was positively correlated with CBL (R = 0.34, *P <*0.0001), VEGFA (R = 0.25, *P <*0.0001), CDH1 (R = 0.33, *P <*0.0001) and FN1 (R = 0.13, *P <*0.05) (Figure 4B). Consistent with PPI network analysis, functional enrichment clustering of 7 nodes shown 9 GO terms are identified to be significant, which containing protein tyrosine kinase activity, positive regulation of cell migration, hepatocyte growth factor receptor signaling pathway, extracellular exosome, epidermal growth factor receptor binding, cytoplasmic vesicle, chemoattractant activity, cell adhesion molecule binding, and activation of MAPK activity (P value <0.05 and -log_10_(P value)>1.301) (Figure 4C). Additionally, Kyoto Encyclopedia of Genes and Genomes (KEGG) analysis was performed to enrich the pathways and found that these nodes have a positive correlation with Ras signaling pathway, Proteoglycans in cancer, PI3K-Akt signaling pathway, Pathways in cancer, MAPK signaling pathway, HIF-1 signaling pathway, Gastric cancer, Focal adhesion, ERBB signaling pathway, EGFR tyrosine kinase, inhibitor resistance, Cytokine-cytokine receptor interaction and Adherents junction (Figure 4D).

### Gene Set Enrichment Analysis (GSEA) identified HGF and c-MET co-related signaling pathway

**Figure 5:**
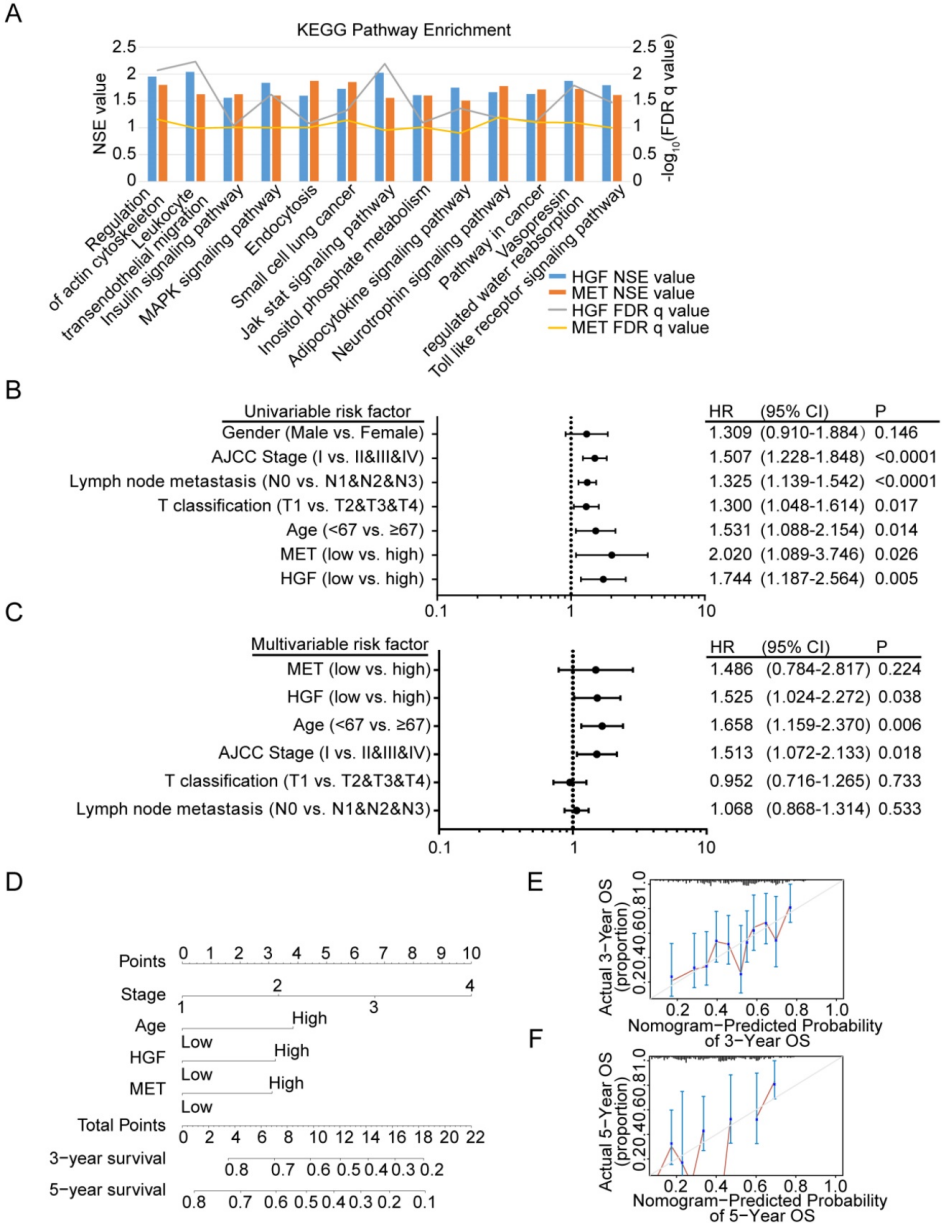
** Diagnostic model based on HGF and c-MET mRNA expression in gastric cancer patients. A.** The diagram shown the 13 shared KEGG pathways enriched by HGF high expressed gastric cancer tissues and c-MET high expressed gastric cancer tissues. **B.** Univariate analysis was performed in gastric cancer cohort based on TCGA database. The bar corresponds to 95% confidence intervals. **C.** Multivariate analysis was performed in gastric cancer cohort based on TCGA database. The bar corresponds to 95% confidence intervals. **D.** The nomogram was utilized by adding up of the points identified on the points scale for each variant. The total points occurred on the bottom scales represent the probability of 3- and 5-year survival. **E&F.** The calibration curve for predicting OS at 3- and 5-years in gastric cancer cohort based on TCGA database.

To identify signaling pathways that are uniquely activated by HGF-c-MET genes, GSEA between low and high HGF and c-MET expression dataset based on TCGA database was conducted. Notably, the optimal cutoff point of HGF and c-MET mRNA expression were calculated based on the X-tile software, which could produce the optimal value to predict survival time. We chose the enriched signaling pathways based on their normalized enrichment score (NSE>1) and False defection rate (FDR<0.05), and found that 45 signaling pathways enriched in the high expression of HGF group, 65 signaling pathways enriched in the high expression of c-MET group, and 13 common signaling pathways enriched in both HGF and c-MET high expression groups. As shown in Figure 5A, MAPK signaling pathway, pathway in cancer, and toll like receptor signaling pathway, and so on, were enriched in HGF and c-MET high expression phenotype.

### Diagnostic model based on HGF and c-MET mRNA expression in gastric cancer patients

We further investigated the prognostic value of HGF and c-MET via using the TCGA database. The univariate analysis revealed that HGF and c-MET high mRNA expression correlated significantly with a poor overall survival (HGF: hazard ratio [HR]: 1.744; 95% confidence interval [CI]: 1.187-2.564; *P =*0.005; c-MET: hazard ratio [HR]: 2.020; 95% confidence interval [CI]: 1.089-3.746; *P =*0.026). Other clinical pathologic variable factors correlated with poor survival included advanced AJCC stage, advanced lymph node metastasis, higher T stage, and older age (Figure 5B), moreover, the results of multivariate analysis shown that HGF remain independently associated with overall survival, with a HR of 1.525 (95% CI: 1.024-2.272, *P =*0.038), along with c-MET, age, AJCC stage, T classification and lymph node metastasis (Figure 5C). To generate a more accurate predictive model, the independent prognostic risk factors in gastric cancer dataset based on TCGA database were utilized to establish a prognostic nomogram. The C-index for overall survival prediction of the formulated nomogram was 0.643 (95% CI: 0.591-0.695; *P <*0.0001), as shown in Figure 5D, the nomogram which constructed based on selected risk factors with hazard ratios could predict the probability of survival through adding up the scores identified on the points scale for every risk factor, and the total score projected to the bottom scale indicated the probability of 3- and 5- year survival. The calibration plot for the probability of 3- and 5- year survival fitted well between the prediction by nomogram and actual observation (Figure 5E & F).

## Discussion

Our study focused on the integrative analyses of HGF and c-MET, and there was consistency between HGF and c-MET in terms of survival prediction, KEGG pathway enrichments and expression level in gastric cancer tissues and peritumor ones.

Previous study have reported that high HGF level in pre-operative serum represent the invasive growth of tumor foci and c-MET positive immunostaining in gastric cancer tissues indicate the tumor with larger diameter, advanced lymphatic vessel invasion and lymph node metastasis 24, the above conclusion was supported by the outcome of our previous work and our colleagues, both of which shown HGF enhance tumorigenesis of gastric cancer via a paracrine pattern 2, 3, 25. We found that HGF-c-MET are enriched in MAPK signaling pathway, PI3K-Akt signaling pathway, JAK-Stat3 signaling pathway and biological process of positive regulation of cell migration, and these analyses had been validated in our prior study, which found PI3K/Akt and ERK1/2 signaling pathway participated in the process of HGF-dependent vascularization in gastric cancer and Jak2/Stat3 signaling pathway involved in the process of HGF-dependent migrant and invasive promotion of gastric cancer.

The correlation between infiltrated immune cells and HGF/c-MET in gastric cancer was analyzed, and results indicated that HGF are positively correlated with CD8+ T cell, CD4+ T cell, macrophage, neutrophil and dendritic cells, which mean that HGF might serve as an immune-positive stimulus via promoting the recruitment of dendritic cells, T lymphocytes, macrophage and neutrophil. Studies found that HGF itself could control the migration of T lymphocytes, induce the expansion of all blood cell type precursors, counteract the anti-inflammatory function of TGF-β, and have a defensive role against inflammation and fibrosis 26. While the c-MET itself could be seen as a tumor-associated antigen (TAA) by CD8 cytotoxic T cells and this function activated immune system against cancer cells that c-MET high expressed 27. Considering the function of HGF-c-MET in angiogenesis formation, and the fact that pro-angiogenetic signals were immunosuppressive, many scholars hypothesized there is a direct link between HGF-c-MET induced angiogenesis, its signals, and the immune suppression of the tumor microenvironment in HGF-c-MET positive tumors 26.

Overexpression of HGF mRNA and/or protein, MET mRNA and c-MET protein have been recorded in 20-30%, and 40-70% samples of gastric cancer patients, respectively 14, 28, which were higher than the proportion of gastric cancer dataset based on TCGA database. Among the Chinese gastric cancer patients, 6% of c-MET genes were amplified and 13% of proteins were overexpressed 15. The c-MET inhibitor, such as Su11274, monotherapy or combined with irinotecan have achieved positive outcomes, other c-MET inhibitor, such as KRC-408, KRC-00715, Simm 530 and multitargeted kinase inhibitor T-1840383 effectively suppressed the proliferation of gastric cancer cells and growth of tumor xenograft 15, besides, a multinational phase 2 study shown the monoclonal antibody which binds the MET-ligand HGF (Rilotumumab) combined with epirubicin, cisplatin, and capecitabine (ECX) could make benefit for MET-positive advanced oesophagogastric cancer patients 29. Thus, the HGF-c-MET signaling pathway is an emerging and very interesting druggable target in gastric cancer, and the gate of molecularly stratified drug development in gastric cancer have opened, the development of medicine and technology might provide new opportunity for the precision treatment of gastric cancer.

## Conclusions

The integrative analyses of HGF and c-MET in this study have been undertaken and found that both of HGF and c-MET high expressed gastric cancer patients have less optimistic prognosis, HGF and c-MET mRNA are higher expressed in gastric cancer tissues rather than those in peritumor ones, GSEA identified the common signaling pathway shared by HGF and c-MET, which could predict the potential mechanism of HGF/c-MET in gastric cancer and nomogram have been constructed, 3-year survival and 5-year survival possibility of gastric cancer patients could be predicted based on the expression level of HGF and c-MET, AJCC stage and Age, such as the patient who have high expressed HGF and c-MET, high Age level and AJCC stage 4, the 3-year survival possibility is less than 20% and 5-year survival possibility is less than 10%.

## Abbreviations

GC: gastric cancer
HGF: hepatocyte growth factor
RTK: receptor tyrosine kinase
TCGA: The Cancer Genome Atlas
HPA: Human Protein Atlas
DAVID: Database for Annotation, Visualization, and Integrated Discovery
KEGG: Kyoto Encyclopedia of Genes and Genomes
TIMER: Tumor Immune Estimation Resource
GSEA: Gene set enrichment analysis
NES: normalized enrichment score
TAA: tumor-associated antigen
ECX: epirubicin, cisplatin, and capecitabine
